# Impact of methane-mitigating concentrate feed on milk production, composition, and udder health in Holstein cows

**DOI:** 10.1007/s44463-025-00033-w

**Published:** 2026-03-27

**Authors:** SangHoon Lee, KyoungBo Ko, GwangHeun Kim, Jong-Eun Park, YounChul Ryu

**Affiliations:** Division of Biotechnology, SARI,, Jeju National University, Jeju City, Republic of Korea

**Keywords:** Methane-mitigating concentrate feed, Enteric methane mitigation, Dairy cows, Milk production, Milk composition, Milk urea nitrogen (MUN), Somatic cell count (SCC), Sustainable dairy production, Greenhouse gas emissions, Commercial farm conditions

## Abstract

Enteric methane emissions from dairy cattle contribute substantially to agricultural greenhouse gas output. Although feed-based mitigation strategies have shown promise in controlled research environments, their effects under commercial farm conditions remain insufficiently studied. This field-based study evaluated the associations between methane-mitigating concentrate (MM) feed and milk yield, milk composition, and udder health indicators in Holstein cows across three commercial farms in Jeju, South Korea. A total of 400 lactating cows were monitored over 47 months (January 2021–November 2024) as the farms transitioned from conventional concentrate feed to MM feed. Mixed models and stepwise regression analyses were used to assess the independent effects of MM feeding while accounting for farm, parity, and seasonal variation. MM feeding did not significantly affect overall milk yield or milk fat content. However, MM feeding was associated with lower milk protein, solid non-fat (SNF), and milk urea nitrogen (MUN) concentrations, along with a modest increase in somatic cell count (SCC). These associations varied among farms and parities, indicating that the effects of MM feed are influenced by management and physiological factors rather than a single dietary component. Stepwise regression retained MM feeding as a significant explanatory variable for SNF, MUN, and SCC, but not for milk yield or fat content. These findings indicate that MM feed can be implemented under commercial farm conditions without compromising milk yield, while influencing nitrogen-related milk traits and udder health indicators. Further studies incorporating direct methane measurements and mechanistic evaluation of rumen fermentation and nitrogen utilization are needed to determine the environmental implications of MM feeding.

**Supplementary Information:**

The online version contains supplementary material available at 10.1007/s44463-025-00033-w.

## Introduction

Climate change represents one of the most pressing global challenges of our time. The Intergovernmental Panel on Climate Change (IPCC) emphasizes the importance of limiting global temperature increases to well below 2 °C above pre-industrial levels. The agriculture, forestry, and other land-use sectors account for approximately 23% of global anthropogenic greenhouse gas emissions. Notably, the livestock sector is a significant source of non-CO₂ gases, particularly methane and nitrous oxide (IPCC, 2022).

Dairy cows are significant contributors to enteric methane emissions. According to the IPCC’s 2006 guidelines, a typical dairy cow releases approximately 128 kg of methane annually, which is more than double the emissions from feedlot beef cattle, averaging approximately 53 kg. These elevated emission levels in dairy cows primarily result from their increased dry matter intake and continuous rumen activity associated with lactation. Methane possesses a global warming potential that is 28 times greater than that of carbon dioxide over a 100-year period (IPCC, 2014). Therefore, reducing methane emissions alone is projected to lower peak global temperatures by up to 0.5 °C by 2050 (Shindell et al., 2012).

Consequently, several governments and international organizations have established policy frameworks and technological initiatives aimed at mitigating methane emissions from livestock. For instance, the European Union has implemented the “2030 Climate Target Plan” and “EU Methane Strategy (COM[2020] 663),” both of which promote robust monitoring systems and the utilization of methane-reducing feed additives, such as 3-nitrooxypropanol (3-NOP) and *Asparagopsis* spp. Similar initiatives have been observed in other regions, including North America, Oceania, and parts of Asia, often embedded within broader climate-smart agriculture strategies.

Feed-based mitigation strategies have become particularly appealing owing to their practicality and cost-effectiveness. Numerous studies indicate that 3-NOP can reduce enteric methane emissions by 24–40% without negatively impacting milk production (Haisan et al., 2014). Additionally, *Asparagopsis* spp., a red seaweed used as a dietary supplement, has demonstrated methane reductions exceeding 80% in controlled research trials (Roque et al., 2021). However, most available data have been derived from tightly controlled experimental settings, leading to a limited understanding of how these interventions perform under commercial farm conditions, where factors such as herd management and climate introduce considerable variability.

This study aimed to address this research gap by evaluating commercially formulated methane-mitigating concentrate (MM) feed under practical field conditions. Specifically, we assessed its associations with key milk production and composition traits, including yield, fat content, protein levels, solid non-fat (SNF) content, somatic cell count (SCC), and milk urea nitrogen (MUN), in Holstein cows managed on commercial dairy farms. The analysis also examined whether the effects of MM feed were influenced by contextual factors such as farm practices, parity, and seasonal variation. Ultimately, this study provides field-based evidence regarding the effects of MM feed on milk composition and udder health under real commercial conditions, offering practical insight into the implementation of MM feeding strategies in dairy production.

## Materials and methods

### Experimental animals and farm management

This study involved a total of 400 lactating Holstein cows from three commercial dairy farms (Farms A, B, and C) located in Jeju City, South Korea. Farm A housed 90 cows with an average parity of 1.8 ± 1.05 and an average of 218.7 ± 141.4 days in milk (DIM); Farm B included 153 cows (parity: 2.5 ± 1.42; DIM: 223.4 ± 144.2); and Farm C comprised 157 cows (parity: 2.41 ± 1.31; DIM: 219.1 ± 146.2). All farms adhered to established herd management protocols, encompassing housing, health monitoring, and milking procedures. The cows were provided with a total mixed ration (TMR) formulated to satisfy the nutritional requirements of high-producing lactating dairy cows, in accordance with the Korean Feeding Standard for Dairy Cattle (National Institute of Animal Science, 2022). TMR was administered twice daily at 06:00 and 18:00. Ingredient compositions and feeding amounts are listed in Table 2. The cows were provided *ad libitum* access to clean drinking water and mineral blocks.

## Feeding strategy timeline and treatment groups

Various feeding strategies were implemented across the farms over time, as summarized in Table 1 and illustrated in Supplementary Fig. 1. Each farm transitioned from standard concentrate feeds to MM feeds at distinct intervals throughout the study period (January 2021 to November 2024). Two commercially manufactured MM feeds were used: Methane Solution™ (Type I; CJ Feed & Care, Republic of Korea; registration No. EEGIR0247) and Custom MaxGreen™ (Type II; Cargill Agri Purina, Republic of Korea; registration No. CCBUR0406). Nutrient composition data for both MM feeds were obtained directly from the manufacturers and are presented in Table 1 as the nutrient composition of the complete MM concentrate feeds. Farm A utilized a standard concentrate feed (Normal I) from January 2021 to December 2022, followed by MM feed Type I from January 2023 onward. Farm B employed Normal I from January 2021 to December 2022, transitioned to MM feed Type I from January to April 2023, and subsequently adopted MM feed Type II from May to November 2024. Farm C utilized Normal II from January 2021 to December 2022 and transitioned to MM feed Type II from January 2023 until the conclusion of the study in November 2024. This staggered feed adoption facilitated an assessment of both spatial (farm-level) and temporal (year/month) variations in response to MM feeding strategies.

**Table 1:** Ingredient composition of the total mixed ration

	Normal feed I and MM feed (type I) (kg/cow/day)	Normal feed II and MM feed (type II) (kg/cow/day)
Normal or MM concentrate feed*	9.8	9.8
Alphacorn	—	2
Milkgen Top	—	0.3
Cottonseed	—	2.8
Beet pulp	1.4	2.3
Oat Hay	3.3	—
Protein supplement	2.0	—
Energy supplement	1.5	—
Supplementary feed	1.8	—
Ryegrass silage (35% moisture)	7	6.67
Alfalfa	2.5	2.5
Triticale	—	3.17
Water	10.0	1.5
Yeast-based probiotic	0.1	0.1
Limestone	—	0.05
Salt	—	0.04
Sodium bicarbonate	0.2	0.2
Total	39.6	31.12

The detailed composition of the methane-mitigating concentrate (MM) feed is proprietary and has not been fully disclosed due to confidentiality. Two additive types were employed: Type I included a yeast-based additive (0.2%), diallyl disulfide (DADS, 0.15%) derived from garlic, and condensed tannins (0.1%), while Type II contained a commercial essential oil mixture (Agolin^®^, 0.2%)

## Feed composition and methane mitigation additives

The TMR comprised both normal and MM feeds, supplemented with forage and other ingredients such as ryegrass silage, beet pulp, alfalfa, triticale, and cottonseed. The nutritional profiles of the concentrate feeds are detailed in Table 2, with all values expressed on a dry matter basis. The MM feeds incorporated methane-mitigating additives, the complete composition of which is proprietary and not fully disclosed. Two additive formulations were used: Type I, containing a yeast-based additive (0.2%), garlic-derived diallyl disulfide (0.15%), and condensed tannins (0.1%); and Type II, containing a commercial blend (Agolin^®^, 0.2%).

**Table 2:** Nutritional composition and physical form of the normal and methane-mitigating concentrate feeds

	Normal feed I	MM feed I	Normal feed II	MM feed II
Feed form	Pellet, crumble			
Feed type	Compound feed for dairy cattle			
Target usage	For cows producing > 40 kg/day			
Crude protein (%)	21	21	20	20
Crude fat (%)	≤ 4.0	≤ 4.0	≤ 3.5	≤ 3.5
Crude ash (%)	≥ 15.0	≥ 15.0	≥ 10.0	≥ 10.0
Crude fiber (%)	≥ 12.0	≥ 12.0	≥ 15.0	≥ 15.0
Calcium (%)	≥ 0.8	≥ 0.8	≥ 1.0	≥ 1.0
Phosphorus (%)	≥ 1.0	≥ 1.0	≥ 1.2	≥ 1.2
TDN (%)	74	74	75	75
Methane-mitigating additive	—	Type I	—	Type II

Abbreviations: MM, methane-mitigating concentrate; TDN, Total Digestible Nutrients

All values are expressed on a dry matter basis

The detailed composition of the methane-mitigating concentrate (MM) feed is proprietary and has not been fully disclosed due to confidentiality. Two additive types were employed: Type I included a yeast-based additive (0.2%), diallyl disulfide (DADS, 0.15%) derived from garlic, and condensed tannins (0.1%), while Type II contained a commercial essential oil mixture (Agolin^®^, 0.2%)

### Statistical analysis

Statistical analyses were conducted using SAS software (version 9.4; SAS Institute Inc., Cary, NC, USA). Data were analyzed to evaluate the effects of feed group (normal, MM), farm (A, B, or C), parity (1–8), and month (1–12) on milk yield and compositional traits, including milk fat, protein, SNF, SCC, and MUN levels.

General linear models (GLM procedure) were employed to estimate the least squares means (LSMeans) and standard errors for each factor independently. Multiple comparisons were adjusted using Tukey’s honest significant difference test, with statistically significant differences among factor levels indicated by superscript letters (*p* < 0.05).

To assess the combined effects and potential interactions of feed group with contextual variables (farm practices, parity, and month), a multifactorial GLM was utilized, incorporating main effects and two-way interaction terms. This approach enabled the determination of whether the impact of MM feed was dependent on the production environment or physiological status.

In addition, linear mixed-effects models were used to further examine the interactions between feed group and farm, parity, and month while accounting for repeated measurements. Individual cows were included as a random effect to control for within-animal correlation over time, and the resulting least squares means and standard errors were plotted.

Finally, stepwise regression modeling was conducted using the GLMSELECT procedure to identify the most parsimonious set of predictors for each trait, guided by the Schwarz Bayesian Criterion (BIC). Adjusted R² and delta BIC values were employed to assess model performance and the contribution of feed group as an explanatory variable. SCC was log₁₀-transformed prior to analysis to satisfy normality assumptions.

## Results and discussion

### Descriptive comparison of milk traits between normal and MM feed

**Fig. 1:**
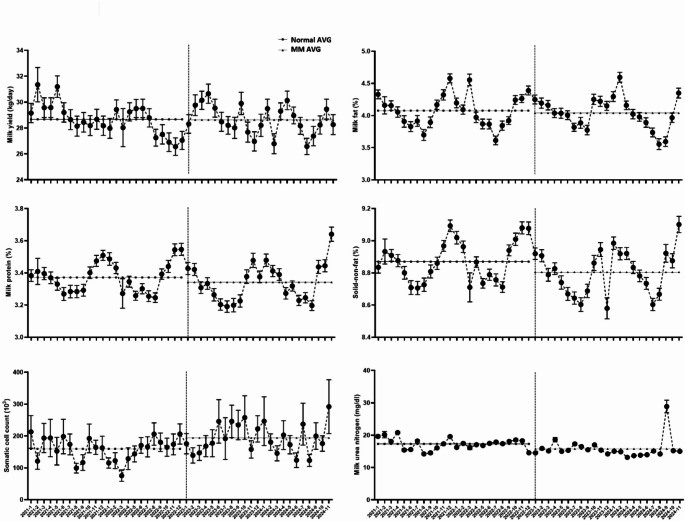
Descriptive comparison of milk production and composition traits by dietary treatment (normal vs. methane-mitigating concentrate (MM) feed). The figure presents monthly arithmetic mean values and standard errors for milk yield (kg/day), milk fat (%), milk protein (%), solid non-fat (%), somatic cell count (10³ cells/mL), and milk urea nitrogen (mg/dL). The plotted data suggest that milk protein, solid non-fat, and milk urea nitrogen were lower in the MM feed group, whereas somatic cell count was slightly elevated. Symbols indicate group means: squares (■) for the normal group and triangles (▲) for the MM feed group. The vertical dotted line denotes the onset of MM feed implementation in January 2023

Figure 1 presents the monthly mean values and standard deviations for milk yield and compositional traits, including milk protein, milk fat, SNF, SCC, and MUN levels, categorized by feed treatment (normal vs. MM) over the measurement period. MM feed was introduced in January 2023, and the data collected cover both the pre- and post-intervention periods. While no formal statistical tests were performed for this figure, it was designed to provide an overview of temporal trends and seasonal variations. The observed patterns indicate that cows on the MM feed exhibited slightly lower average levels of milk protein, SNF, and MUN than those on the normal feed. Additionally, a modest increase in SCC was noted in the MM feed group. Importantly, MUN concentrations were consistently lower in MM-fed cows, suggesting a potential influence of feed formulation on nitrogen metabolism. Although the observed differences are visually apparent, their biological relevance may be minimal and should be interpreted cautiously. Notably, these compositional traits are known to fluctuate with the seasons, and similar trends have been documented in previous studies examining environmental effects on milk yield and quality (Kadzere et al., 2002; Tao & Dahl, 2013; West, 2003).

## Fixed and interaction effects on milk traits

Table 3 presents the least squares means (LSMeans) for milk yield and compositional traits, analyzed via individual fixed effects, including feed group, farm practices, parity, and month. This analysis employed separate GLMs to evaluate the independent contributions of each factor, under the premise that environmental, physiological, and seasonal variations significantly influence lactation outcomes. Previous studies have consistently demonstrated that these variables are major determinants of milk production and composition (Bernabucci et al., 2010; West, 2003).

**Table 3:** Least squares means of milk yield and composition traits by feed group, farm, parity, and month

	Milk yield (kg/day)	Milk fat (%)	Milk protein (%)	SNF (%)	SCC (103)	MUN (mg/dl)
*Feed*						
Normal (*n* = 291)	28.53 (0.59)	4.00 (0.06)	3.27^a^ (0.03)	8.64^a^ (0.03)	213.02^b^ (33.66)	16.99^a^ (0.37)
MM (*n* = 301)	28.50 (0.60)	3.96 (0.06)	3.24^b^ (0.03)	8.57^b^ (0.03)	254.05^a^ (34.02)	15.51^b^ (0.37)
*p* value	0.8907	0.1102	0.0117	< 0.0001	0.0008	< 0.0001
*Farm*						
Farm A (*n* = 90)	27.66^a^ (0.63)	4.38^a^ (0.06)	3.17^b^ (0.03)	8.44^c^ (0.03)	225.71^a^ (35.67)	16.58^a^ (0.39)
Farm B (*n* = 153)	27.78^b^ (0.59)	3.92^b^ (0.06)	3.30^a^ (0.03)	8.74^a^ (0.03)	294.06^b^ (33.63)	16.48^a^ (0.36)
Farm C (*n* = 157)	30.12^a^ (0.60)	3.63^c^ (0.06)	3.28^b^ (0.03)	8.64^b^ (0.03)	180.83^c^ (34.12)	15.69^b^ (0.37)
*p* value	< 0.0001	< 0.0001	< 0.0001	< 0.0001	< 0.0001	< 0.0001
*Parity*						
1 (*n* = 222)	26.42^c^ (0.18)	4.24^a^ (0.02)	3.37^a^ (0.01)	8.91^a^ (0.01)	132.36^c^ (9.96)	17.06^a^ (0.11)
2 (*n* = 175)	28.80^c^ (0.21)	4.15^b^ (0.02)	3.39^a^ (0.01)	8.85^b^ (0.01)	155.97^c^ (11.93)	16.75^a^ (0.13)
3 (*n* = 134)	30.00^b^ (0.25)	4.06^c^ (0.02)	3.34^b^ (0.01)	8.75^c^ (0.01)	186.08^c^ (14.19)	16.06^b^ (0.16)
4 (*n* = 90)	30.05^ab^ (0.33)	4.06^c^ (0.03)	3.29^cd^ (0.02)	8.71^c^ (0.02)	310.04^b^ (18.65)	15.35^c^ (0.21)
5 (*n* = 46)	31.05^a^ (0.47)	4.04^cd^ (0.05)	3.22^d^ (0.02)	8.62^d^ (0.03)	292.76^b^ (26.56)	16.01^bc^ (0.30)
6 (*n* = 15)	27.83^c^ (0.74)	3.75^d^ (0.07)	3.21^d^ (0.04)	8.51^e^ (0.04)	438.04^a^ (41.90)	15.35^bc^ (0.47)
7 (*n* = 4)	28.82^bc^ (3.49)	3.88^d^ (0.34)	3.13^d^ (0.18)	8.16^e^ (0.19)	98.89^c^ (197.74)	18.41^a^ (2.20)
8 (*n* = 1)	25.18^c^ (2.95)	3.65^d^ (0.29)	3.09^d^ (0.15)	8.34^e^ (0.16)	254.15^bc^ (167.17)	15.02^c^ (1.74)
*p* value	< 0.0001	< 0.0001	< 0.0001	< 0.0001	< 0.0001	< 0.0001
*Month*						
1 (*n* = 332)	28.58^abcd^ (0.68)	4.17^ab^ (0.07)	3.33^b^ (0.03)	8.70^ab^ (0.04)	243.47 (38.66)	15.92^de^ (0.42)
2 (*n* = 351)	28.73^abcd^ (0.70)	4.21^a^ (0.07)	3.31^bc^ (0.04)	8.69^ab^ (0.04)	204.27 (39.52)	16.21^cde^ (0.43)
3 (*n* = 352)	29.64^ab^ (0.70)	4.05^bc^ (0.07)	3.25^cd^ (0.04)	8.63^bc^ (0.04)	212.81 (39.44)	15.07^e^ (0.43)
4 (*n* = 348)	29.88^a^ (0.67)	3.93^cd^ (0.07)	3.22^de^ (0.03)	8.61^c^ (0.04)	225.52 (38.19)	17.28^b^ (0.42)
5 (*n* = 349)	29.74^a^ (0.68)	3.86^d^ (0.07)	3.19^ef^ (0.03)	8.53^d^ (0.04)	217.02 (38.50)	15.03^e^ (0.42)
6 (*n* = 353)	28.83^abc^ (0.68)	3.81^de^ (0.07)	3.14^fg^ (0.03)	8.49^de^ (0.04)	238.95 (38.56)	15.40^de^ (0.42)
7 (*n* = 351)	27.83^cde^ (0.68)	3.73^e^ (0.07)	3.13^g^ (0.03)	8.43^e^ (0.04)	246.03 (38.74)	16.98^bc^ (0.42)
8 (*n* = 347)	27.61^de^ (0.68)	3.67^e^ (0.07)	3.12^g^ (0.03)	8.44^e^ (0.04)	220.48 (38.75)	15.38^de^ (0.42)
9 (*n* = 353)	28.38^bcd^ (0.68)	3.72^e^ (0.07)	3.23^de^ (0.03)	8.60^c^ (0.04)	235.72 (38.74)	19.17^a^ (0.42)
10 (*n* = 354)	27.90^cde^ (0.67)	4.08^b^ (0.07)	3.31^bc^ (0.03)	8.67^ab^ (0.04)	252.16 (38.13)	16.52^cde^ (0.42)
11 (*n* = 354)	27.47^e^ (0.68)	4.21^a^ (0.07)	3.43^a^ (0.03)	8.79^a^ (0.04)	252.54 (38.65)	16.34^cde^ (0.42)
12 (*n* = 298)	27.65^de^ (0.71)	4.29^a^ (0.07)	3.37^ab^ (0.04)	8.69^ab^ (0.04)	253.46 (40.54)	15.70^de^ (0.44)
*p* value	< 0.0001	< 0.0001	< 0.0001	< 0.0001	0.8032	< 0.0001

Abbreviations: MM, methane-mitigating concentrate; MUN, Milk Urea Nitrogen; SCC, Somatic Cell Count; SNF, Solid Non-Fat

Least squares means (standard error) were estimated from separate general linear models for each factor

Different letters (a-g) indicate significant differences within factor levels (Tukey-adjusted, *p* < 0.05)

Across feed groups, MM-fed cows exhibited significantly lower levels of milk protein, SNF, and MUN than those fed the normal feed. In contrast, SCC was slightly elevated in the MM feed group. These commercially formulated MM concentrate feeds are formulated to modify rumen fermentation characteristics and may affect nutrient utilization efficiency. Previous studies have reported that methane-mitigating feed additives can alter ruminal hydrogen dynamics and nutrient digestibility, indicating shifts in fermentation patterns (van Gastelen et al., 2020). However, the magnitude and consistency of these responses vary depending on diet composition, dosage, and farm management conditions, as highlighted in recent reviews and meta-analyses evaluating methane-mitigating feeding strategies (Beauchemin et al., 2022; Kebreab et al., 2023).

Significant farm-level differences (*p* < 0.0001) were observed across all traits. Farm C demonstrated the highest average milk yield and the lowest SCC, while Farm A exhibited the highest milk fat percentage. These variations likely reflect differences in farm-level management practices, feed formulations, and microclimatic conditions. Parity exerted a strong effect on all traits (*p* < 0.0001). Milk yield steadily increased from the first to the fifth lactation, reaching a plateau or declining thereafter. Conversely, milk protein, SNF, and MUN displayed a gradual reduction with increasing parity. These trends are consistent with the physiological demands of repeated lactation, which have been associated with cumulative metabolic stress and declining hepatic and mammary efficiency (Berry et al., 2006; Gross, 2022; Hurley et al., 2012). Older cows often experience a deeper negative energy balance in early lactation and exhibit reduced nutrient-partitioning efficiency, partially explaining the lower concentrations of protein-rich milk components and urea nitrogen. The observed parity-related decline in MUN may also reflect diminished ruminal nitrogen recycling or altered microbial activity (Arndt et al., 2015). Milk composition parameters demonstrated significant seasonal variation. Monthly averages revealed noticeable reductions in milk protein and SNF during the summer months (June–August), possibly because of heat stress-induced alterations in feed intake and rumen function. These findings align with prior research on thermal stress and lactation performance (Kadzere et al., 2002; West, 2003). In contrast, SCC remained relatively stable across months (*p* = 0.8032), suggesting that mammary gland health is more strongly influenced by parity structure or farm hygiene than by seasonal climate alone.

While the univariate models presented in Table 3 provide insights into individual fixed effects, Table 4 expands the analysis by incorporating a multifactorial GLM with two-way interactions among feed groups and contextual factors, including farm practices, parity, and month. This approach examines the context-dependent effects of MM feeding.

**Table 4:** Multifactorial general linear model results for main and interaction effects on milk traits

	Milk yield	Milk fat	Milk protein	SNF	SCC	MUN
*Feed*	0.6844	< 0.0001	0.0011	< 0.0001	0.0003	< 0.0001
*Farm*	< 0.0001	< 0.0001	< 0.0001	< 0.0001	< 0.0001	< 0.0001
Feed * Farm	0.3479	< 0.0001	0.0003	< 0.0001	0.1252	< 0.0001
*Feed*	0.1224	0.2168	0.0098	< 0.0001	0.3743	0.0033
*Parity*	< 0.0001	< 0.0001	< 0.0001	< 0.0001	< 0.0001	< 0.0001
Feed * Parity	0.0943	0.0669	< 0.0001	< 0.0001	< 0.0001	0.0454
*Feed*	0.6276	0.0036	0.0042	< 0.0001	0.017	< 0.0001
*Month*	< 0.0001	< 0.0001	< 0.0001	< 0.0001	0.7647	< 0.0001
Feed * Month	0.0532	< 0.0001	0.0844	< 0.0001	0.9584	< 0.0001

p-values from a multifactorial general linear model evaluating main effects and two-way interactions (Feed × Farm, Feed × Parity, Feed × Month)

Abbreviations: MUN, Milk Urea Nitrogen; SCC, Somatic Cell Count; SNF, Solid Non-Fat

In the Feed × Farm model, MM feeding exerted significant main effects on milk fat, milk protein, SNF, SCC, and MUN levels, all with p-values less than 0.001. However, no significant effect on milk yield was noted (*p* = 0.6844). The interaction between Feed and Farm was significant for all traits, except milk yield and SCC, indicating that the influence of the MM feed on milk composition varied by farm for most parameters. Regarding parity (Feed × Parity model), MM feeding was significantly associated with lower levels of milk protein (*p* = 0.0098), SNF (*p* < 0.0001), and MUN (*p* = 0.0033). In contrast, milk yield (*p* = 0.1224), milk fat (*p* = 0.2168), and SCC (*p* = 0.3743) had no significant effects on milk yield. The interaction between Feed and Parity was considerably significant for milk protein, SNF, SCC, and MUN (all *p* < 0.0001), indicating that the effects of feed group varied across different stages of lactation. In terms of seasonal influence (Feed × Month model), MM feeding significantly affected all milk compositional traits, except milk yield (*p* = 0.6276). Unlike other models, the Feed × Month interaction was exclusively significant for SNF and MUN (both *p* < 0.0001), suggesting relatively stable monthly differences in these specific parameters among the feed groups. Only the interaction between feed and month for SCC was not significant (*p* = 0.9584), indicating that temporal fluctuations in mammary gland health may not be strongly linked to MM feeding.

Collectively, these interaction effects underscore the importance of evaluating feeding strategies within the variability of commercial systems. As emphasized by Dijkstra et al. (2025), context-dependent responses are critical when transitioning from experimental to field-scale applications of methane mitigation technologies. The current findings highlight that MM feeding may influence milk quality traits variably depending on farm conditions and cow physiology, which must be accounted for in any implementation or policy development.

Taken together, these findings demonstrate that while MM feed does not negatively affect milk volume, its compositional impacts—particularly on SNF, MUN, and SCC—are context-sensitive and should be interpreted within the broader production environment. Further studies integrating animal-level physiology, rumen fermentation dynamics, and farm-level nutritional strategies are required to clarify the underlying mechanisms.

## Group-Wise Mixed-model comparisons

**Fig. 2:**
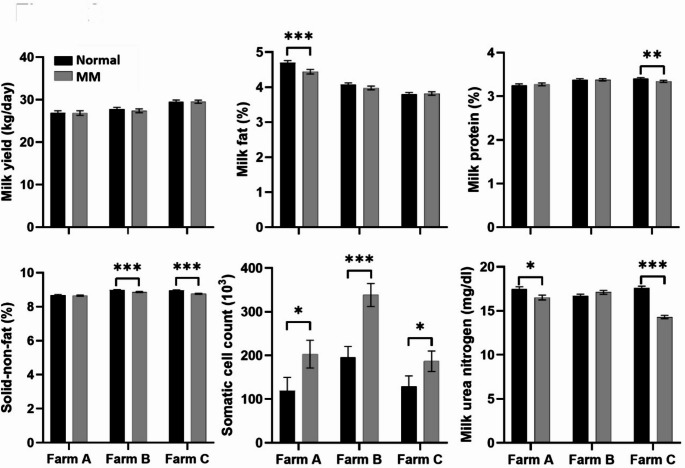
Comparisons of milk production and composition traits by feed group across farms (A, B, and C). Least squares means for milk yield (kg/day), milk fat (%), milk protein (%), solid non-fat (%), milk urea nitrogen (mg/dL), and somatic cell count (10³ cells/mL) are shown for cows fed normal and methane-mitigating concentrate (MM) feed. Error bars represent standard errors of the mean. Black bars represent the normal feed group, and grey bars represent the MM feed group. Asterisks indicate statistically significant differences between feed groups within each farm: * *p* < 0.05, ** *p* < 0.01, *** *p* < 0.001

**Fig. 3:**
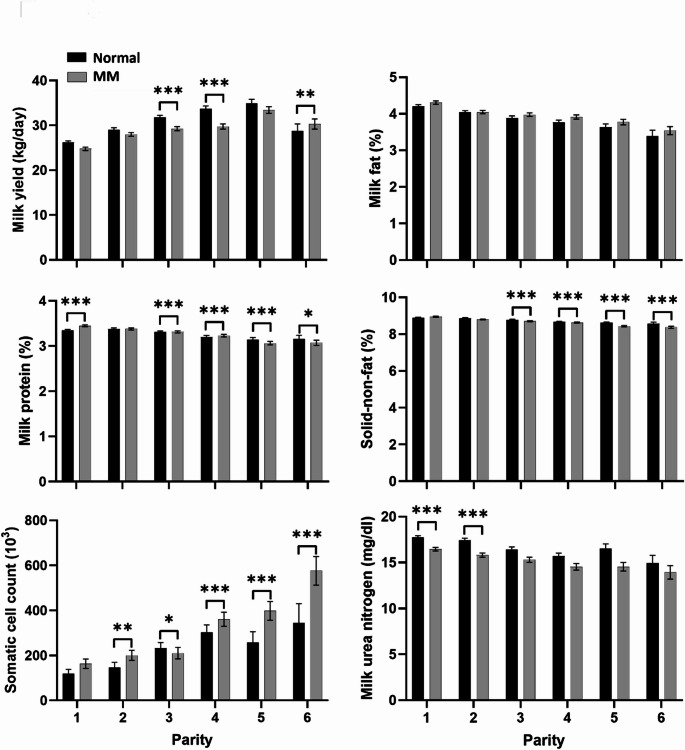
Comparisons of milk production and composition traits by feed group across parity levels (1st to 8th parity). Least squares means for milk yield (kg/day), milk fat (%), milk protein (%), solid non-fat (%), milk urea nitrogen (mg/dL), and somatic cell count (10³ cells/mL; log-transformed) are shown for cows fed normal and methane-mitigating concentrate (MM) feed. Error bars represent standard errors of the mean. Black bars represent the normal feed group, and grey bars represent the MM feed group. Asterisks indicate statistically significant differences between feed groups within each parity: * *p* < 0.05, ** *p* < 0.01, *** *p* < 0.001

**Fig. 4:**
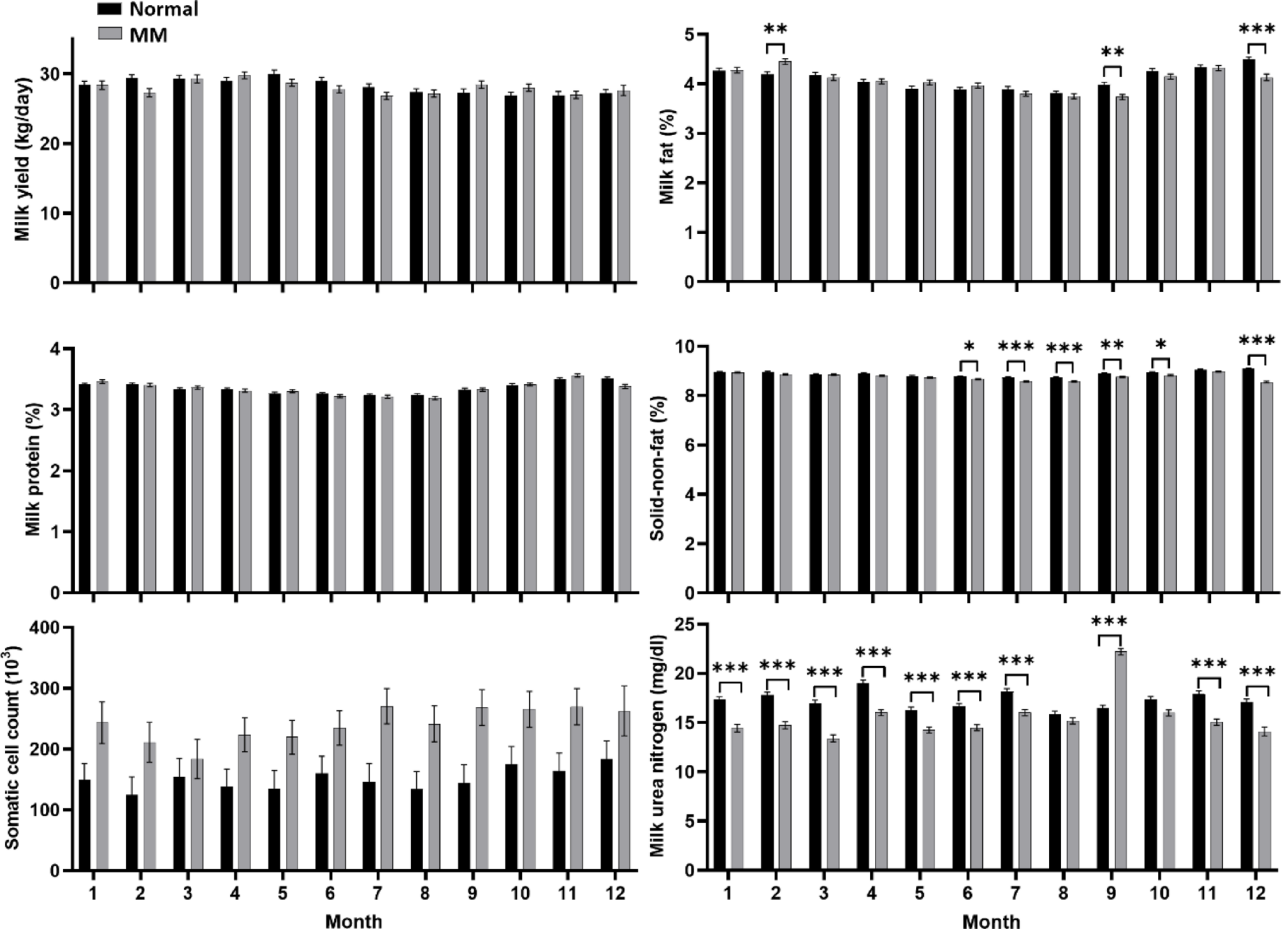
Comparisons of milk production and composition traits by feed group across calendar months. Least squares means for milk yield (kg/day), milk fat (%), milk protein (%), solid non-fat (%), milk urea nitrogen (mg/dL), and somatic cell count (10³ cells/mL; log-transformed) are shown for cows fed normal and methane-mitigating concentrate (MM) feed. Error bars represent standard errors of the mean. Black bars represent the normal feed group, and grey bars represent the MM feed group. Asterisks indicate statistically significant differences between feed groups within each month: * *p* < 0.05, ** *p* < 0.01, *** *p* < 0.001

Figures 2, 3 and 4 display the results of linear mixed-model analyses investigating the interactions among feed group and three factors: farm practices, parity, and month. These models accounted for repeated measurements by including cow ID as a random effect, thereby enhancing the accuracy of variability estimates within individual cows over time. As depicted in Fig. 2, comparisons by farm indicated that MM feeding varied with the production environment. Specifically, the impact of feed group exhibited variation across farms, emphasizing the significance of farm-specific conditions in influencing dietary responses.

MM feeding demonstrated distinct effects across farms. Milk fat was significantly lower in MM-fed cows on Farm A (***p < 0.001), while no significant differences were detected on Farms B and C. Milk protein levels were significantly lower in MM-fed cows on Farm C (**p < 0.01) but remained unchanged on Farms A and B. Additionally, SNF content was significantly lower in MM-fed cows on Farms B and C (***p < 0.001), with no significant difference on Farm A. SCC was significantly elevated in the MM feed group across all farms (*p < 0.05 on Farms A and C, ***p < 0.001 on Farm B), indicating a consistent, albeit moderate, increase in SCC. In contrast, MUN levels were significantly lower in MM-fed cows on Farms A and C (*p < 0.05 and ***p < 0.001, respectively), with no significant changes on Farm B.

These findings reveal farm-specific responses to MM feeding, particularly concerning traits related to milk quality and nitrogen metabolism. The observed differences potentially reflect variations in forage quality, environmental conditions, or baseline herd management practices. The results suggest that the effectiveness of MM feed may depend on baseline nutritional management, forage quality, or hygiene protocols at each farm. This aligns with prior research reporting variable methane mitigation efficacy across commercial operations (Olijhoek et al., 2016).

Figure 3 illustrates the parity-specific effects of the MM feed on six milk traits. Parity 7 was excluded from the analysis owing to insufficient and unbalanced observations, while parity 8 was omitted because of an uneven representation across feed groups.

Milk yield displayed variable responses to MM feeding across different parities. A significant increase in milk yield was noted in parity 6, while parities 3 and 4 exhibited significantly lower milk yields in the MM feed group. No significant differences were observed among feed groups in parities 1, 2, and 5. Although milk fat content did not significantly differ among feed groups across all parities, MM-fed cows demonstrated a slight numerical increase in milk fat at higher parities. Milk protein content was lower in cows fed MM feed across most parities. Milk protein concentration can vary due to nutritional and physiological factors rather than being determined by a single feed effect (Huhtanen et al., 2015). Therefore, the parity-dependent differences observed in this study should be interpreted cautiously. For SNF, a consistent decline was observed in the MM feed group across all parities, particularly in parities 3–6. SCC was elevated in MM-fed cows in parities 2, 4, 5, and 6, with a slight increase also noted in parity 3. Although the absolute differences were moderate, these findings suggest a potential parity-specific sensitivity of mammary health responses to MM feeding. Milk MUN levels were consistently lower in the MM feed group across all parities, although statistical significance was not achieved beyond parity 2. This trend may indicate reduced nitrogen excretion or altered rumen microbial efficiency in response to dietary changes.

These findings partially align with prior reports indicating that increasing parity is associated with diminished metabolic adaptability, liver function, and immune responsiveness (Gross, 2022; Hurley et al., 2012). The observed decline in MUN levels corroborates earlier research suggesting that nitrogen recycling efficiency decreases with age (Arndt et al., 2015). However, considering that most referenced studies utilized experimental diets not specifically designed for methane mitigation, the parity-specific trends observed in our MM feeding trial provide new, contextually relevant evidence.

Figure 4 presents the monthly least squares means and standard errors for milk traits comparing normal and MM feed from January to December. Overall, no significant differences in milk protein content were detected among the feed groups across all months. In contrast, SNF was significantly lower in the MM group during months 6–10 and again in December, suggesting a potential sensitivity to feed treatment during specific seasons. Regarding MUN, the MM feed group generally exhibited lower values; nevertheless, no significant differences were observed in August or October. SCC remained relatively stable and did not display a clear pattern of seasonal variation among the feed groups.

These findings indicate that the impact of MM feeding on milk composition may fluctuate over time; nonetheless, the monthly trends do not entirely align with classical expectations regarding seasonal or thermal stress responses. Previous studies have demonstrated that high ambient temperatures can negatively affect rumen fermentation and milk solids (Kadzere et al., 2002; Tao & Dahl, 2013). In contrast, the current results reveal a more intricate pattern. Notably, SNF exhibited a progressive decline from summer through winter in the MM feed group, rather than a sharp, heat-related decrease. Similarly, MUN differences persisted across most months, with a few exceptions, suggesting that factors beyond heat stress—such as individual cow variability, forage quality, and feed intake—may influence seasonal responses to MM feed. The observed monthly variations imply that the effects of MM feeding may not exclusively emanate from seasonality but are rather modulated by a combination of farm-level, environmental, and physiological factors.

Collectively, these stratified analyses underline the imperativeness of contextualizing nutritional interventions within the inherent biological variability associated with lactational and temporal factors. While parity-related differences in MM feed responses can be interpreted within established physiological frameworks, the inconsistent month-to-month patterns highlight the impact of environmental and management-related variables. Future research that incorporates detailed feed composition data, animal-level health indicators, and farm-specific management practices may yield deeper insights into the mechanisms driving these heterogeneous responses.

### Evaluation of fixed effects on milk traits using Stepwise regression analysis

To assess the relative importance of potential predictors and ascertain whether MM feeding independently influences milk traits while accounting for structural variables, we employed stepwise regression modeling utilizing the GLMSELECT procedure. Model selection was guided by the Schwarz Bayesian Criterion, which prioritizes simplicity by imposing a penalty for model complexity. Candidate explanatory variables included feed group, farm practices, parity, and month. Additionally, the SCC variable was log-transformed prior to modeling to ensure compliance with normality assumptions.

The results summarized in Table 5 indicate that farm and parity consistently emerged as key explanatory variables across all traits, reinforcing their established significance in shaping production outcomes under field conditions. Conversely, MM feeding was not identified as a statistically significant factor for milk yield, fat, or protein, suggesting that MM feeding may not independently influence these traits when accounting for other structural effects. However, feed group was retained in the final SNF, MUN, and log-transformed SCC models, each demonstrating strong statistical support (*p* < 0.0001). These traits also led to modest improvements in model fit, as reflected by negative *Δ*BIC values, particularly for MUN (*Δ*BIC = − 50) and SCC (*Δ*BIC = − 30) indicating that the inclusion of MM feeding improved model performance.

**Table 5:** Summary of Stepwise regression results for milk traits based on the bayesian information criterion

Dependent Variable	Feed group effect	Farm effect	Parity effect	Month effect	Adjusted *R*^2^	Direction of feed group effect	ΔBIC
Milk yield	No	Yes	Yes	No	0.0656	-	0
Milk fat	No	Yes	Yes	Yes	0.1969	-	0
Milk protein	No	Yes	Yes	Yes	0.0732	-	0
SNF	Yes (*p* < 0.0001)	Yes	Yes	Yes	0.1483	Decrease in MM feed	-20
SCC	Yes (*p* < 0.0001)	Yes	Yes	No	0.0888	Increase in MM feed	-30
MUN	Yes (*p* < 0.0001)	Yes	Yes	Yes	0.0847	Decrease in MM feed	-50

Abbreviations: BIC, Bayesian Information Criterion; MUN, Milk Urea Nitrogen; MM, methane-mitigating concentrate; SCC, Somatic Cell Count; SNF, Solid Non-Fat

SCC: Somatic Cell Count (log₁₀-transformed)

Adjusted R^2^: Coefficient of determination adjusted for number of predictors

*Δ*BIC: Difference in BIC between the baseline model (excluding feed group) and the full model (including feed group) as a predictor. A negative *Δ*BIC value indicates improved model fit with the inclusion of feed group

“Yes” indicates that the variable was retained in the final model based on BIC minimization

“Feed group Effect”: Result of stepwise selection based on BIC. “No” indicates feed group was not selected

p-values are only reported when feed group was retained (*p* < 0.05)

The MM feed was associated with lower SNF and MUN values. However, these associations should not be interpreted as causal. Milk composition traits can vary with changes in nitrogen use efficiency, which is known to respond to dietary protein level and ruminal fermentability. Previous studies have shown that altering dietary crude protein level or nitrogen utilization efficiency can influence milk composition responses, including MUN (Castillo et al., 2000; Sinclair et al., 2014). The reduction in MUN observed in MM-fed cows may reflect differences in nitrogen utilization rather than increased nitrogen allocation to milk protein synthesis. In addition, farm and parity contributed substantially to variation in the model, indicating that milk composition responses are context-dependent and influenced by multiple physiological and environmental factors.

In contrast, the model also identified a modest yet statistically significant increase in SCC within the MM-fed group. While this result does not imply clinical mastitis, it may reflect subtle shifts in immune status, inflammatory responses, or other stress-related physiological responses. Dietary components that alter rumen fermentation can influence systemic immunity by affecting ruminal pH, microbial turnover, and the release of lipopolysaccharides, which act as inflammatory triggers and increase SCC even in the absence of infection (Khafipour et al., 2009; Patra & Yu, 2012). Considering the multifactorial nature of SCC, this finding should be interpreted with caution. SCC may also be influenced by parity distribution, seasonal effects, or hygiene inconsistencies rather than a direct feed-related effect. Supporting this interpretation, recent studies utilizing other methane-reducing additives, such as *Asparagopsis taxiformis* and 3-NOP, have reported similar inconsistencies in SCC outcomes (Haisan et al., 2014; Roque et al., 2019).

The overall explanatory power of the models remained moderate, with adjusted R² values ranging from 0.0656 (milk yield) to 0.1969 (milk fat). These results reflect the complex nature of milk production, which is influenced by both physiological and environmental factors. The additional unexplained variance likely stems from unmeasured variables, such as genetic background, individual feed intake, cow-level energy balance, and microbial variation in the rumen. Future model refinement may benefit from integrating these covariates to capture the biological complexity of lactation responses more comprehensively. While the adjusted R² values appear modest, this is expected in large, field-based multi-farm datasets where milk traits are influenced by numerous uncontrolled biological and environmental factors. In inferential models such as GLMSELECT, the primary goal is to determine whether fixed effects (feed, farm, parity, and month) have meaningful associations with the outcomes rather than to maximize predictive accuracy. Therefore, adjusted R² should not be interpreted as a measure of effect validity.

Even though feed group was not retained as a predictor of milk yield, the retained effects of MUN and SCC indicate that MM feeding strategies may influence nitrogen metabolism and immune-related indicators. These findings are broadly consistent with earlier meta-analyses, which have demonstrated context-dependent outcomes in response to methane mitigation diets (Dijkstra et al., 2025).

Overall, the results suggest that MM feed modulates milk composition in subtle yet detectable ways, particularly regarding nitrogen-related traits, without compromising milk volume. Nevertheless, the observed changes in MUN, SNF, and SCC levels highlight the necessity for balanced assessments that consider environmental benefits alongside potential trade-offs in milk quality and udder health. Further research, including detailed feed characterization, longitudinal monitoring of inflammatory markers, and cow-level physiological profiling, is essential to elucidate these dynamics and facilitate broader adoption of methane-reducing feed interventions in commercial dairy herds.

## Conclusions

This field-based study evaluated the use of MM feed under commercial dairy production conditions. MM feed was associated with changes in milk composition, particularly lower milk protein, SNF, and MUN values, while milk yield remained unaffected. These responses varied among farms and parities, suggesting that the effects of MM feed are context dependent and influenced by both environmental and physiological factors rather than a single dietary component.

Although MM feed is formulated with the intention of reducing enteric methane emissions, methane emissions were not directly measured in this study. Therefore, the results should not be interpreted as evidence of methane reduction. Instead, our findings demonstrate that MM feed can be implemented in commercial dairy operations without compromising milk production performance, while influencing nitrogen-related milk traits.

Future studies that include direct methane measurements and controlled evaluations of rumen fermentation characteristics and nitrogen utilization efficiency are required to clarify the biological mechanisms underlying these compositional responses and to verify the environmental mitigation potential of MM feed strategies.

## Supplementary Information

Below is the link to the electronic supplementary material.

Supplementary Material 1

Supplementary Material 2

## Data Availability

The data that support the findings of this study are available from the corresponding author upon reasonable request.
