# Supplementary material for: Impact of methane-mitigating concentrate feed on milk production, composition, and udder health in Holstein cows
Source: Food Sci Anim Resour. 2026 Mar 27;46(1):49. doi: 10.1007/s44463-025-00033-w (PMC13031564; doi:10.1007/s44463-025-00033-w)

**Supplementary figure legend**

SFigure 1.

Timeline of feed strategy implementation across three participating dairy farms (A, B, and C) from January 2021 to November 2024. Each row represents a farm, with different shading patterns indicating the type of feed administered over time. Farm A fed a conventional concentrate diet (Normal I) until December 2022 and adopted low-methane (LM) feed Type I beginning in January 2023. Farm B transitioned from Normal I (January 2021–December 2022) to LM feed I (January–April 2024), followed by LM feed II (May–November 2024). Farm C used Normal II until December 2022 and switched to LM feed II starting in January 2023. This staggered introduction of LM diets allowed for the analysis of both farm-specific and time-dependent responses to methane mitigation feeding strategies.


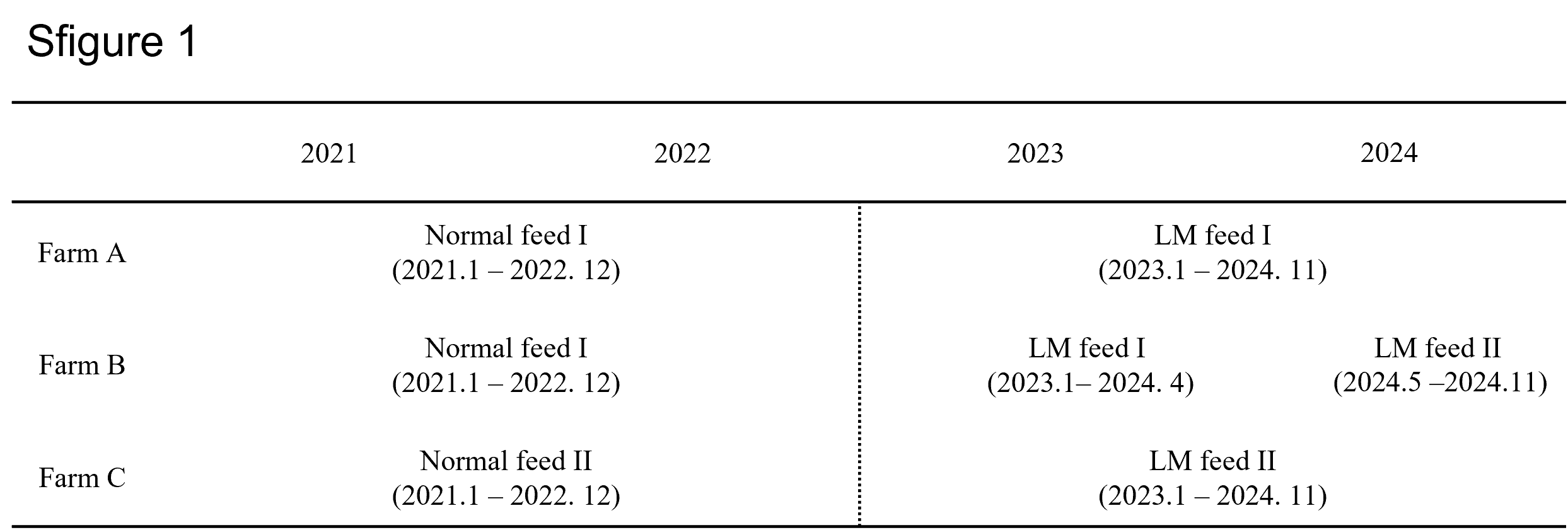

Supplement: Supplementary file 1 — Supplementary Material 1 [file 44463_2025_33_MOESM1_ESM.docx]
