# Supplementary material for: Impact of methane-mitigating concentrate feed on milk production, composition, and udder health in Holstein cows
Source: Food Sci Anim Resour. 2026 Mar 27;46(1):49. doi: 10.1007/s44463-025-00033-w (PMC13031564; doi:10.1007/s44463-025-00033-w)
Supplement: Supplementary file 2 — Supplementary Material 2 [file 44463_2025_33_MOESM2_ESM.docx]

**References**

2023. Climate change 2022 - mitigation of climate change: Working group iii contribution to the sixth assessment report of the intergovernmental panel on climate change. Cambridge University Press, Cambridge.

Arndt C, Powell JM, Aguerre MJ, Crump PM, Wattiaux MA. 2015. Feed conversion efficiency in dairy cows: Repeatability, variation in digestion and metabolism of energy and nitrogen, and ruminal methanogens. J Dairy Sci 98:3938-3950.

Beauchemin KA, Ungerfeld EM, Abdalla AL, Alvarez C, Arndt C, Becquet P, Benchaar C, Berndt A, Mauricio RM, Mcallister TA, Oyhantçabal W, Salami SA, Shalloo L, Sun Y, Tricarico J, Uwizeye A, De Camillis C, Bernoux M, Robinson T, Kebreab E. 2022. Invited review: Current enteric methane mitigation options. Journal of Dairy Science 105:9297-9326.

Bernabucci U, Lacetera N, Baumgard LH, Rhoads RP, Ronchi B, Nardone A. 2010. Metabolic and hormonal acclimation to heat stress in domesticated ruminants. Animal 4:1167-1183.

Berry DP, Macdonald KA, Penno JW, Roche JR. 2006. Association between body condition score and live weight in pasture-based holstein-friesian dairy cows. Journal of Dairy Research 73:487-491.

Castillo AR, Kebreab E, Beever DE, France J. 2000. A review of efficiency of nitrogen utilisation in lactating dairy cows and its relationship with environmental pollution. Journal of Animal and Feed Sciences 9:1-32.

Dijkstra J, Bannink A, Congio GFS, Ellis JL, Eugène M, Garcia F, Niu M, Vibart RE, Yáñez-Ruiz DR, Kebreab E. 2025. Feed additives for methane mitigation: Modeling the impact of feed additives on enteric methane emission of ruminants—approaches and recommendations. Journal of Dairy Science 108:356-374.

Gross JJ. 2022. Limiting factors for milk production in dairy cows: Perspectives from physiology and nutrition. J Anim Sci 100.

Haisan J, Sun Y, Guan LL, Beauchemin KA, Iwaasa A, Duval S, Barreda DR, Oba M. 2014. The effects of feeding 3-nitrooxypropanol on methane emissions and productivity of holstein cows in mid lactation. J Dairy Sci 97:3110-3119.

Hristov AN. 2024. <em>invited review</em>: Advances in nutrition and feed additives to mitigate enteric methane emissions. Journal of Dairy Science 107:4129-4146.

Huhtanen P, Cabezas-Garcia EH, Krizsan SJ, Shingfield KJ. 2015. Evaluation of between-cow variation in milk urea and rumen ammonia nitrogen concentrations and the association with nitrogen utilization and diet digestibility in lactating cows. Journal of Dairy Science 98:3182-3196.

Hurley W, Bionaz M, Loor JJ. 2012. Milk protein synthesis in the lactating mammary gland: Insights from transcriptomics analyses. In Milk protein. Hurley W (ed.). IntechOpen, Rijeka.

Intergovernmental Panel on Climate C. 2014. Climate change 2014: Mitigation of climate change. Cambridge University Press, Cambridge, UK and New York, NY, USA.

Kadzere CT, Murphy MR, Silanikove N, Maltz E. 2002. Heat stress in lactating dairy cows: A review. Livestock Production Science 77:59-91.

Kebreab E, Bannink A, Pressman EM, Walker N, Karagiannis A, Van Gastelen S, Dijkstra J. 2023. A meta-analysis of effects of 3-nitrooxypropanol on methane production, yield, and intensity in dairy cattle. Journal of Dairy Science 106:927-936.

Khafipour E, Krause DO, Plaizier JC. 2009. Alfalfa pellet-induced subacute ruminal acidosis in dairy cows increases bacterial endotoxin in the rumen without causing inflammation. Journal of Dairy Science 92:1712-1724.

National Institute of Animal S. 2022. Korean feeding standard for dairy cattle: Fourth edition. National Institute of Animal Science, Wanju, Korea.

Olijhoek DW, Hellwing ALF, Brask M, Weisbjerg MR, Højberg O, Larsen MK, Dijkstra J, Erlandsen EJ, Lund P. 2016. Effect of dietary nitrate level on enteric methane production, hydrogen emission, rumen fermentation, and nutrient digestibility in dairy cows. J Dairy Sci 99:6191-6205.

Patra AK, Yu Z. 2012. Effects of essential oils on methane production and fermentation by, and abundance and diversity of, rumen microbial populations. Appl Environ Microbiol 78:4271-4280.

Roque BM, Salwen JK, Kinley R, Kebreab E. 2019. Inclusion of asparagopsis armata in lactating dairy cows’ diet reduces enteric methane emission by over 50 percent. Journal of Cleaner Production 234:132-138.

Roque BM, Venegas M, Kinley RD, De Nys R, Duarte TL, Yang X, Kebreab E. 2021. Red seaweed (asparagopsis taxiformis) supplementation reduces enteric methane by over 80 percent in beef steers. PLoS One 16:e0247820.

Shindell D, Kuylenstierna JCI, Vignati E, Van Dingenen R, Amann M, Klimont Z, Anenberg SC, Muller N, Janssens-Maenhout G, Raes F, Schwartz J, Faluvegi G, Pozzoli L, Kupiainen K, Höglund-Isaksson L, Emberson L, Streets D, Ramanathan V, Hicks K, Oanh NTK, Milly G, Williams M, Demkine V, Fowler D. 2012. Simultaneously mitigating near-term climate change and improving human health and food security. Science 335:183-189.

Sinclair KD, Garnsworthy PC, Mann GE, Sinclair LA. 2014. Reducing dietary protein in dairy cow diets: Implications for nitrogen utilization, milk production, welfare and fertility. Animal 8:262-274.

Tao S, Dahl GE. 2013. Invited review: Heat stress effects during late gestation on dry cows and their calves. Journal of Dairy Science 96:4079-4093.

Van Gastelen S, Dijkstra J, Binnendijk G, Duval SM, Heck JML, Kindermann M, Zandstra T, Bannink A. 2020. 3-nitrooxypropanol decreases methane emissions and increases hydrogen emissions of early lactation dairy cows, with associated changes in nutrient digestibility and energy metabolism. Journal of Dairy Science 103:8074-8093.

West JW. 2003. Effects of heat-stress on production in dairy cattle. Journal of Dairy Science 86:2131-2144.
